# Shaping Consumer Perceptions of Genetically Modified Foods: The Influence of Engineering, Science, and Design Signifiers in Packaging Disclosure Statements

**DOI:** 10.3390/foods14060909

**Published:** 2025-03-07

**Authors:** Bryan F. Howell, Ellyn M. Newcomb, D. Wendell Loh, Asa R. Jackson, Michael L. Dunn, Laura K. Jefferies

**Affiliations:** 1 Department of Design, Brigham Young University, Provo, UT 84602-3113, USA; 2 Department of Nutrition, Dietetics & Food Science, Brigham Young University, Provo, UT 84602-3113, USA; emnewcomb@hotmail.com (E.M.N.); wendellloh@gmail.com (D.W.L.); michael_dunn@byu.edu (M.L.D.); laura_jefferies@byu.edu (L.K.J.); 3 Lab for Social Design, Kolding School of Design, 6000 DK Kolding, Denmark; asajackson100@gmail.com

**Keywords:** nudging GM food attitudes, human-centered food disclosure statements, improving BE food perceptions, bioengineered food perceptions, National Bioengineered Food Disclosure Standard perceptions

## Abstract

Genetically modified (GM) foods have existed for decades, and governments internationally have legislated packaging disclosure statement language that typically incorporates the words genetic, modified, and organism. In 2018, the United States implemented the National Bioengineered Food Disclosure Standard (NBFDS) and introduced the term *Bioengineered* (BE) into GM disclosure language to help clarify consumer uncertainty regarding GM foods. Since then, the US consumer attitudes, perceptions, and knowledge of genetically modified foods remain negative, reflecting a contaminated interaction. Current mandated disclosure labels, utilizing engineering and science-based signifiers, are associated with this negative interaction. This research assesses whether food disclosure labels based on the signifier *Design*, unassociated with current contaminations, can positively impact the consumer perception of GM foods compared to the negatively contaminated science and engineering signifiers currently used. Two online studies of 1931 participants analyzed GM/BE food disclosure labels comparing four existing and six newly created engineering and science-based signifiers against four new design-based signifiers across fifteen attributes, including Price, Purchase Likelihood, Environmental Impact, Fair Trade, Safety, Nutrition, Healthfulness, Quality, Eating Experience, Comforting, Inviting, Frightening, Understandable, Ethical, and Sustainable. Across both studies, design-related labels consistently outperformed traditional engineering/science-based terms in fostering positive perceptions. However, even the best-performing labels did not fully overcome the entrenched skepticism associated with GM foods, underscoring the need for complementary strategies beyond linguistic changes.

## 1. Introduction

Genetically modified (GM) foods have been available for public purchase since the early 1990s, and soon after, governments began regulating GM foods’ relationships with consumers through food label disclosure statements, as they are a primary means of informing the public about GMOs [1]. The word choices made by regulating entities for disclosure statements vary somewhat but typically include the words genetic, modified, and organism. For example, in 2000, Japan passed a labeling standard that required food products to be labeled as *Genetically Modified*, *Genetically Modified Organisms Not Segregated*, or *Not Genetically Modified* [2]. Soon after that, the European Union (EU) also elected to use the terms *Genetically Modified* and *Genetically Modified Organism* (GMO) in their 2001/2003 legislative and regulatory directives [3]. Other intergovernmental agencies such as the Organization for Economic Co-operation and Development (OECD) and the Food and Agriculture Organization of the United Nations (FAO) employed yet another GM term, *Genetic Biotechnology*, in their GM food communications [4,5]. Recognizing the value in disclosure statements used elsewhere, consumer advocacy groups, non-profit organizations, and food manufacturing entities in the United States of America began requesting that the GM nature of foods be stated on product labels [6,7,8,9]. In 2013, the State of Vermont passed the USA’s first GM food disclosure statement law. The House of Representatives introduced House Bill 112, requiring that qualifying raw commodities and processed foods containing genetically modified material be labeled with one of the following disclosure statements: *Partially Produced With Genetic Engineering*, *May Be Produced With Genetic Engineering*, or *Produced With Genetic Engineering* [10]. Soon afterward, similar bills and disclosure statement laws were passed in Connecticut [11] and Maine [12], which required the term *Produced With Genetic Engineering* to be conspicuously disclosed on retail foods. Noticeably, these laws introduced the word engineering into known GM food disclosure statement conventions and eliminated the words modified and organism used internationally.

To standardize GM labeling requirements across states, in 2016, the 114th United States Congress amended the Agricultural Marketing Act of 1946 by enacting Senate Bill 764, authorizing the Secretary of Agriculture to establish a National Bioengineered Food Disclosure Standard (NBFDS) for foods that are genetically modified or contain GM ingredients [13]. The resulting federal standard, issued in December 2018, diverged from known word conventions used internationally and in state laws by incorporating the term *Bioengineered* (BE) to label GM foods. This new Bioengineering Disclosure Statement (BDS) standard subjugated the word genetic, universally used to describe GM foods, as an element defining the new term *Bioengineered* [14]. Months before the final ruling was issued, the US Department of Agriculture (USDA) Agricultural Marketing Service (AMS) sought public opinion on the standard [15]. One question specifically solicited comments on the adoption of four proposed text disclosure statements: *Bioengineered Food*, *Contains Bioengineered Food Ingredients*, *May Contain Bioengineered Food Ingredients*, and *May Be Bioengineered*. In subsequent public comment summaries published with the Final Rule, AMS stated that some commenters “requested straightforward labeling that would not confuse consumers by using unfamiliar terms”. Many commenters suggested allowing or mandating previously used phrases such as *Genetically Modified Organism*, *GMO*, or *Genetic Engineering*. Other commenters felt that AMS’s departure from historical terminology would confuse consumers and suggested that the NBFDS “clarify the definition of bioengineering to state that it is synonymous with ‘genetic engineering’ or ‘GMO’”. In response to these comments, the AMS stated, “The Secretary believes that the [*Bioengineered*] language used by Congress in the amended (2016) Act clearly and accurately describes the technology and provides consumers with the information they desire”. They also stated that the AMS will engage in outreach and education to provide information about the new disclosure term [15].

The discussion of public comments in the Final Rule exposes multiple issues, including deliberations leading up to the text selection for the mandatory disclosure statement. First, no disclosure options included the term *Genetic* as historically used by state legislators and international trading partners, even though the public highlighted this discrepancy multiple times. Second, the *Bioengineered* disclosure language ultimately adopted—though consistent with the legislation—was much less familiar to the public. Though unstated, legislators and regulators may have felt that the longstanding use of the terms GM, GMO, and similar biotechnology language by activist opponents of GM foods could have negatively affected how US consumers perceive and interpret those terms, making it necessary to make a clean break by adopting the term *Bioengineered* on US food labels. Finally, in intentionally introducing this new vocabulary into the disclosure statement, the USDA also recognized the need to educate the public on new terminology, laying out for itself the responsibility of a consumer education campaign. Since the 1990s, GM foods have received public backlash among a vocal group of opponents, making the adoption of biotechnology in food production slow and consumers wary [16,17]. Consumers have associated labels indicating the presence of GM ingredients with reduced food value [18] and, conversely, associate *non-GMO* labeling with greater value and consumer willingness to pay [19,20]. The Center for Food Safety (CFS) filed lawsuits challenging the Final Rule as currently implemented [21,22,23], alleging that the term *Bioengineered* is misleading and allows manufacturers to conceal or avoid labeling GM foods or foods produced with GM ingredients because the regulation is restrictive and unclear [24]. Other law critics have also stated that the NBFDS scope is too narrow and defective [25]. Despite the publication of the Final Rule in 2018 and its subsequent implementation, consumer perceptions of GM foods remain negative, creating social sludge, a friction that makes it harder to obtain outcomes that will help people be better off [26]. While consumer interaction is multidimensional, it is largely cognitive and includes conative and affective elements [27]. We propose that this complex relationship between consumer perceptions and GM food technology reflects a negatively contaminated interaction, which can lead to object avoidance, devaluation, and misuse [28].

Contamination means making something impure; it deviates from a pure or undefiled ideal state [29]. Contaminated interaction is “the presence of some real or imagined property that alters how a user perceives and engages with a material”. An object can be contaminated through real changes like physical alteration, or through imagined changes resulting from cognitive associations [28]. Objects can have negative, neutral, or positive interactions; they remain neutral until they are positively or negatively contaminated. Contaminated interactions require a meaningful change in an object. Meaning is shaped by individual perception and social context, and evolves with time [30]. Research has linked contamination to crucial areas such as hygiene (health preservation), territory (managing personal space), and utility (how changes in an object affect its value) [31,32]. Negatively contaminated interactions between consumer perceptions and GM-food technology reflect a utilitarian-based value change in the object, i.e., GM foods. Conversely, research also shows that positive interaction contaminations exist and produce the opposite effect by significantly improving the meaning and value of an object [28,31]. For example, objects belonging to famous celebrities are often positively contaminated and have greater fiscal and emotional value and meaning than the identical object owned by a common individual. Importantly, all contaminations occur at the individual level, and impact both consumer markets and business-to-business exchanges. Contamination drivers are influenced by culture and personal experience, and shape meaning and influence consumer–object interactions. The presence of such drivers in the context of GM food lays a foundation for testing hypotheses around contaminated interactions [28,31]. Understanding the negative and positive drivers regarding GM food disclosure label interactions could benefit consumers and industry. The contamination drivers potentially influencing interactions between GM food labels and consumers stem from the multiple disciplines involved. The languages, values, and goals of those disciplines are portrayed in the words used in the mandatory disclosure labels. Pure science is “a method of investigating nature by the experimental method in an attempt to satisfy the need to know”. Applied science is “the use of pure science for some practical human purpose”. Science is the need “to know and do” [33]. Engineering is “the strategy for causing the best change in a poorly understood or uncertain situation within the available resources, by reason”. Engineering is the “ability to distinguish between the true and the false” with “reason” or logic [34].

Consumers seek signs that indicate and explain technology’s purpose. These clues, known as signifiers, play a crucial role in communicating an object’s purpose and use, and are essential for navigating the social and technological landscape of that object [35]. Contemporary GM/BE disclosure labels are composed of science and engineering vocabulary, *genetics*, *bio*, *engineering*, *technology*, and *produced*. These words are signifiers and, whether intentional or incidental, communicate associations, values, and meanings to the consumers who read them. We hypothesize that the disciplinary language in past and current GM food disclosure is negatively contaminated and has contributed to adverse consumer perceptions. Testing new signifiers from the discipline of design, a human-centered discipline associated with both engineering and science, but not commonly used in the GM food lexicon, might foster more favorable consumer attitudes. The word design is broadly defined and includes both processes and outcomes. The process of design is used in both science and engineering, such as designing an experiment or a prototype. Design is also an outcome; it is the result of some kind of “intervention that changes existing conditions into preferred ones”, including, services, procedures, strategies, and policies [36]. The basic principles of design processes and methods focus on the interactions between people and technology. A good design process leads to useful, usable, and desirable product, and experience outcomes, when done well; however, poor design results in frustration and irritating outcomes [37]. As such, the word design in this context is neutrally contaminated. It is unknown if it will produce positive or negative interactions. Design signifies a human-centered process and outcome. The word design is also a companion term, easily combined with the current disclosure word’s *genetics*, *bio*, *engineering*, *technology*, and *produced*. For example, genetically designed, bio-designed, design engineering, designed technology, and design and production are lexically related.

For this study, we assessed whether food disclosure labels based on the neutral word design will positively or negatively impact the consumer perception of GM foods in the United States when compared to the known negatively contaminated science and engineering signifiers currently used. Understanding how disclosure label signifiers positively or negatively contaminate consumer interactions with GM/BE foods could aid stakeholders in positioning their products. Hypothesis 1: Study 1 assessed consumer perceptions of GM/BE foods disclosure labels incorporating the signifier *engineered*, and *genetic*, against labels using the signifier *designed.* We predicted that GM/BE foods labeled with *design* signifiers will demonstrate significantly more positive consumer perceptions than labels with the negatively contaminated *engineering* and *genetic* signifiers. Hypothesis 2: Building on the insights of Study 1, Study 2 assessed whether eight new disclosure terms combining science-, engineering-, and design-related signifiers could improve perceptions across a broader set of attributes. We expected that GM/BE food labels limited to design related signifiers will be positively perceived when compared to labels with signifiers derived from engineering and science.

## 2. Materials and Methods

**Figure 1:**
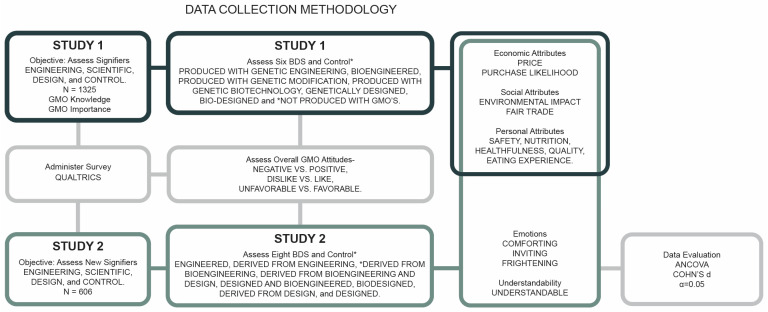
Schematic outline of the data collection methodology. The dark outlined boxes indicate activities in study 1, the light outlined boxes represent common activities in both study 1 and 2, and the medium green outlined boxes indicate activities in study 2. The overlapping boxes indicate shared content in both studies.

This flow diagram provides a visual summary of this multi-stage study to improve understanding. The study schematic (see Figure 1) illustrates the data collection methodology for each stage of the study. The steps for the two studies, depicted in the boxes in Figure 1, include the respective BDS and control terms, and the accompanying question categories. Study 1 assessed general attitudes and knowledge about GMOs, and perceptions of engineering/scientific and design terms in the context of three attribute categories. Study 2 objectives built on Study 1 by assessing combined engineering/scientific and design terms among a broader range of question categories. The validity of this study is demonstrated by the large sample sizes and recruitment of participants through reputable online survey firms. BDSs were compared against control terms, and responses were collected using standardized Likert scales. Written answers were independently reviewed by two researchers. Analysis of Covariance (ANCOVA) and effect sizes using Cohen’s *d* were used to determine the statistical significance and clarity of the practical relevance of findings.

Two studies were conducted using online surveys administered to convenience samples of US residents (Survey 1 n = 1325, Survey 2 n = 606), aged ≥ 18 years, who shared equally in grocery shopping or were the primary household grocery shoppers. Surveys were created and administered using Qualtrics XM^®^ (Qualtrics, L.L.C., Provo, UT, USA) survey software. Participants were recruited using a database from ESOMAR^®^ (ESOMAR, Amsterdam, The Netherlands). Potential participants received an initial online invitation from ESOMAR. They then clicked a uniform resource locator (URL) link, which took them to a pre-screening page to determine eligibility. For both surveys, eligible participants were directed to the study consent form and, following consent, entered the survey. Incomplete survey data were discarded and not used in any analysis. Data were analyzed using Statistical Analysis Software SAS^®^ version 9.4 (SAS Institute Inc., Cary, NC, USA). The independent variables included the disclosure statement, education, age, lifestyle, and self-assessed GMO knowledge. The dependent variables included Consumer Attitudes, Purchase Likelihood, and impact on Price, Fair Trade, Environmental Impact, Safety, Nutrition, Healthfulness, Quality, and Eating Experience.

The average consumer ratings for each question presented in the study were compared statistically using covariance analysis. In Study 1, the average ratings of study participants’ self-assessed GMO knowledge and their quantified knowledge scores were also compared using an analysis of covariance. A post hoc Tukey–Kramer method (α = 0.05) measured all possible pairwise differences of means from the covariance analysis, as this method adjusts for the slight variation in participant sample sizes. The effect size was measured using Cohen’s *d*. No time requirements were implemented to complete the questionnaires. Participants were compensated for their time based on Qualtrics^®^ panel rates. The experiment was approved by the University’s Institutional Review Board (IRB) for Human Subjects (IRB study number E16347).

### 2.1. Study 1: Comparison of US and International BDS Against the Proposed Design BDS

Throughout the remaining manuscript, GM/BE food disclosure labeling statements are referred to as Bioengineered Disclosure Statements or BDS.

#### 2.1.1. Study 1 Participants

Relatively equal gender ratios responded, with nearly half (n = 663, 50.2%) identifying as female and half identifying as male (n = 660, 49.8%). Females varied in age from 18 to 85 years, with a mean of 41.1 years. Males ranged from 18 to 84 years, with a mean of 44.0 years. A stratified sampling method was used to collect a random sample from each geographical region in the United States: Northeast (18.2%), Midwest (21.9%), South (36.5%), and West (23.4%). Current relationship status indicated that 47.8% of the participants were married, 30.6% were never married, 11.1% were divorced, 5.7% were partners, 2.9% were widowed, and 2.0% were separated. The combined household income was an overall mean of USD 46,500. Each participant was randomly assigned a questionnaire for only one of six BDSs or the control term. All seven questionnaires were identically framed and administered to all participants. An equal number of participants (n = 189) completed each questionnaire.

#### 2.1.2. Study 1 Bioengineered Disclosure Statement Selection

Selected BDS science and engineering signifiers came from the US and international legal terms; *Bioengineered*, *Produced with genetic engineering*, *Produced with genetic modification*, and *Produced with genetic biotechnology*. The created BDS design signifiers were *Genetically designed* and *Bio-designed*. It is believed that these terms have not been used publicly in the context of labeling GM/BE foods. The control term was *Not produced with GMOs* (*NGMO*). None of the BDSs were defined for participants. Prior knowledge of these terms was not assessed nor were terms defined, as the emphasis of this study was participant face value reaction to their presence on a food label.

#### 2.1.3. Study 1 Participant GMO Attitudes

The first question asked participants to rate their overall attitudes toward a food product labeled with the particular BDS or control terminology presented. Participants rated their attitudes using three discrete 7-point word-anchor scales where 0 = Negative, Dislike, or Unfavorable, and 7 = Positive, Like, or Favorable, respectively. The midpoint score of 3.5 divided favorable scores from unfavorable scores.

#### 2.1.4. Study 1 Effect of BDSs on Food-Related Attributes

The next questions assessed economic attributes, consisting of Purchase Likelihood and Price; social attributes, consisting of Fair Trade and Environmental Impact; and personal attributes, consisting of Safety, Nutrition, Healthfulness, Quality, and Eating Experience. A definition of Fair Trade was provided: “Fair Trade refers to trade in which fair prices are paid to producers in developing countries”. Participants were not given definitions for any other attributes. This series of questions was worded as follows: for Question 1, “For a given food product that you regularly purchase, how likely would you be to purchase it if its label stated (insert a BDS or the control term)?” or for Questions 2–9, “How do you think the (insert attribute) of the food would be affected if its label stated (insert a BDS or the control statement)?” Responses for attributes were recorded on a discrete 5-point word anchor scale. For Purchase Likelihood, 1 = much less likely, and 5 = much more likely to purchase. For the other attributes, 1 = greatly decrease, and 5 = greatly increase. A score of 3.0 on the rating scale indicates a neutral response for all nine attributes. Ratings between 3.0 and 1.0 indicate a decreasing effect, whereas ratings between 3.0 and 5.0 indicate an increasing effect.

#### 2.1.5. Study 1 GMO Knowledge

To analyze how perceived versus actual participant GMO knowledge might affect their response, another survey question asked, “How knowledgeable do you consider yourself on the topic of GMOs (genetically modified organisms)?” Responses were recorded on a discrete 6-point scale anchored with endpoints where 1 = not at all knowledgeable and 6 = extremely knowledgeable. The next question asked participants to demonstrate their understanding of GMOs by writing a short response to the following question: “In your own words, describe a GMO (Genetically Modified Organism)”. Written responses were evaluated against the standard of the World Health Organization’s definition of genetically modified organisms [38], noting that the written responses may not capture all subjects’ comprehensive understanding. Individual responses were independently reviewed by two researchers knowledgeable in the field, using a set of keywords and the standard definition to assign a numerical score from 1 to 6 on a discrete scale anchored with endpoints ‘No understanding of GMOs’ (1) and ‘Excellent understanding of GMOs’ (6). Significant discrepancies between the reviewers were few and, when identified by individual scores differing by 2 or more points, reviewers arrived at a mutually agreeable rating by averaging their scores.

#### 2.1.6. Study 1 Importance of GMOs

The final question assessed how important the topic of GMOs was to the participant personally. Responses were recorded on a 6-point scale anchored with endpoints 1 = not at all important and 6 = extremely important.

### 2.2. Study 2: Comparison of Eight New BDSs Against Each Other and a Control Statement

#### 2.2.1. Study 2 Participants

Relatively equal gender ratios were surveyed, with 50.7% identifying as female (n = 308), and 49.1% as male (n = 298), and one who chose not to answer. Females varied in age from 18 to 85 years, with a mean of 41.1 years. Males ranged from 18 to 84 years, with a mean of 44.0 years. Regarding Study 1, the same sampling method was used to collect data from each geographical region in the United States. Of the participants, 17.2% were from the Northeast, 21.6% from the Midwest, 45.5% from the South, and 15.7% from the West. On entering the survey, each participant was randomly assigned an identical questionnaire varying by one of the eight newly created BDSs or the control statement.

#### 2.2.2. Study 2 Bioengineered Disclosure Statement Selection

New BDS terms were created to reflect stand-alone and combined disciplinary signifiers. The first category, engineering terms, includes *Engineered* and *Derived from Engineering*. The second category, engineering, science, and design terms, includes *Derived from Bioengineering and Design* and *Designed and Bioengineered*. The third category, science and design terms, includes *Biodesigned* and *Derived from Biodesign*. The fourth category, design terms, includes *Derived from design* and *Designed*. The control term is from the terminology used in US federal regulation [14], *Derived from Bioengineering*. None of the BDSs were defined for participants. Prior knowledge of these terms was not assessed nor were terms defined, as the emphasis of this study was participant face value reaction to their presence on a food label. The word *Produced*, used in Study 1, was replaced with the word *Derived* in Study 2 to match the control term.

#### 2.2.3. Study 2 Participant GMO Attitudes

Questions about overall attitudes for each BDS are identical to that of Study 1.

#### 2.2.4. Study 2 Effect of BDSs on Food-Related Attributes

Questions about economic, social, and personal attributes for each new BDS are identical to that of Study 1. Six additional attributes were assessed in Study 2. The emotion attributes included Inviting, Comforting, and Frightening. The cultural attributes included Ethical and Sustainable. The understandability attribute is Understandability. Like in study 1, the same definition of Fair Trade was provided. Participants were not given definitions for any other attributes. These questions asked participants, “For a given food product that you regularly purchase, how (insert new attribute) is the statement (insert BDS/control) when included on the label?” Responses were presented using a discrete 5-point scale. For five of the six questions, the scale was 1 = not at all and 5 = very. For the sixth question with the attribute Frightening, the scale was reversed, 1 = very frightening, 2 = somewhat frightening, 3 = neither, 4 = somewhat not frightening, and 5 = not at all frightening. For all attributes, a score of 3.0 on the rating scale indicated that participants were neutral in their responses. Ratings between 3.0 and 1.0 indicated a decreasing effect, whereas ratings between 3.0 and 5.0 indicated an increasing effect, except for Frightening, which was reversed.

### 2.3. Reporting Results

To avoid redundancy, only results that emerged as statistically significant are reported, unless otherwise stated. Key Cohen’s size effects are reported in the text. Cohen’s *d* provides a standardized measure of how each label drives perception of the attributes. Positive *d* values indicate favorable associations, while negative *d* values indicate unfavorable associations. Effect size threshold is defined as Negligible (*d* < 0.2), Small (*d* < 0.50), Medium (*d* < 0.80), and Large *d* ≥ 0.80. All the graphs used the same pairwise comparison method. The same letters within attributes indicate no significant difference between the means (*p* > 0.05). Different letters indicate a statistically significant difference between the means.

In Study 1, the letters are Tukey-adjusted, 95% confidence intervals are normal approximations, n = 189 per statement group, and n = 1325. In Study 2, the letters are Tukey-adjusted, 95% confidence intervals are normal approximations, and n = 606. In attitude graphs in Studies 1 and 2, reported means greater than the 3.5 midpoint were rated more favorable, while those less than 3.5 were rated less favorable. For all attribute graphs in Studies 1 and 2, reported means greater than the 3.0 midpoint were rated as increasing, while those less than 3.0 were rated as decreasing.

## 3. Results

### 3.1. Study 1 Results

#### 3.1.1. Study 1 GMO Attitudes

**Figure 2:**
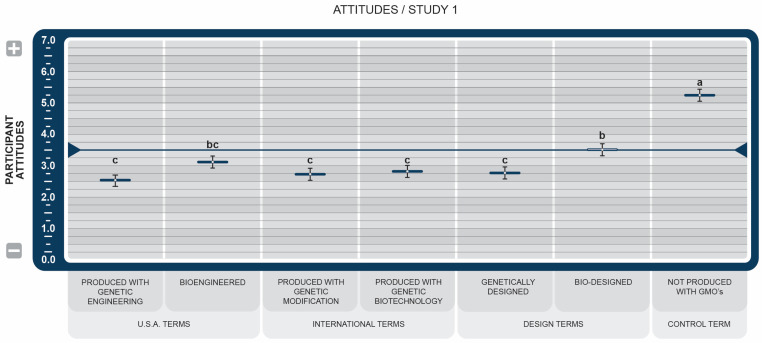
Study 1 effect of Genetically Engineered Disclosure statements on combined participant attitudes (positive/negative, like/dislike, favorable/unfavorable). a–c: Like superscripts represent no significant differences between means (*p* < 0.05).

The results show that the choice of anchor words did not produce significantly different results (*p* < 0.001), indicating that participants did not interpret the three attitude scales differently. Figure 2 illustrates the average participant scores of the combined attitudes for each disclosure statement. All BDSs scored significantly lower in favorability than the control (*p* < 0.043). However, the term *Bio-designed* was the only BDS, excluding the control term, with a score just above the 3.5 midpoint.

#### 3.1.2. Study 1 Effect of BDSs on Food-Related Attributes

Study 1 revealed that engineering/science-based labels are deeply associated with negative consumer attitudes and that design-focused labels evoked positive associations, strongly validating Hypothesis 1. Other than the control term, all BDSs, except *Bio-designed*, had a less than favorable impact on the participant’s perception of all attributes. Bio-designed, with its human-centered associations, had the greatest influence on positive perceptions of these attributes. Consumers appeared to associate most engineering- and science-based terms, including *Genetically Designed* with concerns in all attribute categories.

**Figure 3:**
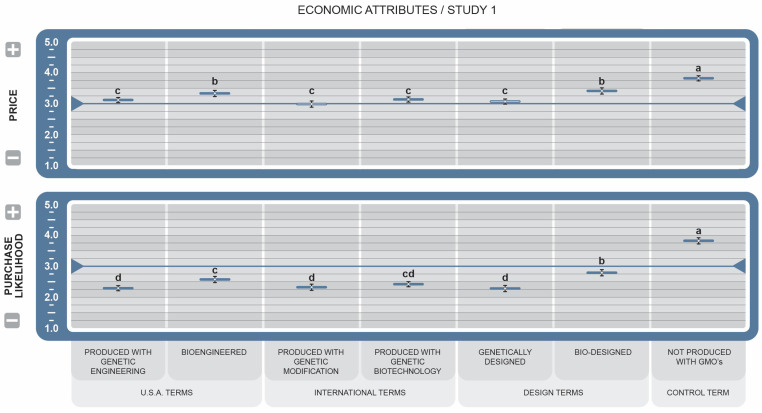
Effect of Genetically Engineered Disclosure statements on economic attributes. Means greater than the 3.0 neutral midpoint were rated as increase/more likely to purchase, while those less than 3.0 were rated as decrease/less likely to purchase. a–d: Like superscripts represent no significant differences between means (*p* < 0.05).

In economic attributes (see Figure 3), food products labeled with the control term (µ = 3.42 ± 0.10, *d* = 12.25) and *Bio-designed* (µ = ±0.10, *d* = 1.57) were the highest scoring and primary positive drivers of Purchase Likelihood but were also viewed as being the highest priced. *Bioengineered* (µ = 3.33 ± 0.10, *d* = 0.58) had a moderate effect on higher pricing, while all remaining BDSs, largely comprised of scientific and engineering language, had large associations with lower pricing, but were less likely to inspire purchase.

**Figure 4:**
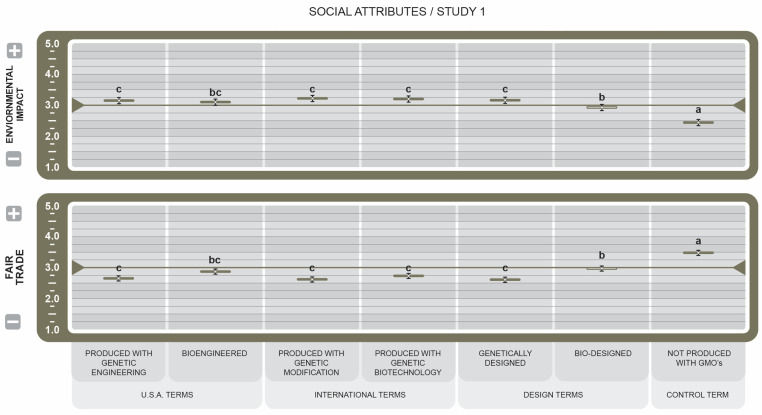
Effect of Genetically Engineered Disclosure statements on participant social attributes. a–c: Like superscripts represent no significant differences between means (*p* < 0.05).

In social attributes (see Figure 4), responses to the Environmental Impact attribute indicate that, unsurprisingly, the control term was associated with a lower environmental impact (µ = 3.56 ± 0.10) and conveyed the strongest ties to eco-friendliness (*d* = 5.98). Similarly, *Bio-designed* also outscored (µ = 2.07 ± 0.10, *d* = 1.01) the other BDSs, which were rated as having a slight positive environmental impact, yet the overall associations between the terms and the environment were negative. Responses to the Fair Trade attribute indicate the control term as the most favorable (µ = 3.48 ± 0.1), with the strongest links to positive fair trade practices (*d* = 6.91). *Bio-designed* (µ = 3.0, *d* = 1.32) and *Bioengineered* (µ = 2.90, *d* = 0.58) were rated at the neutral point, perhaps indicating consumer ambiguity. On the other hand, the BDSs that conveyed unfavorable attitudes about their impact on equitable production and pricing were *Genetically Designed* (µ = 2.61 ± 0.1, *d* = 2.4) and labels using scientific and engineering signifiers.

**Figure 5:**
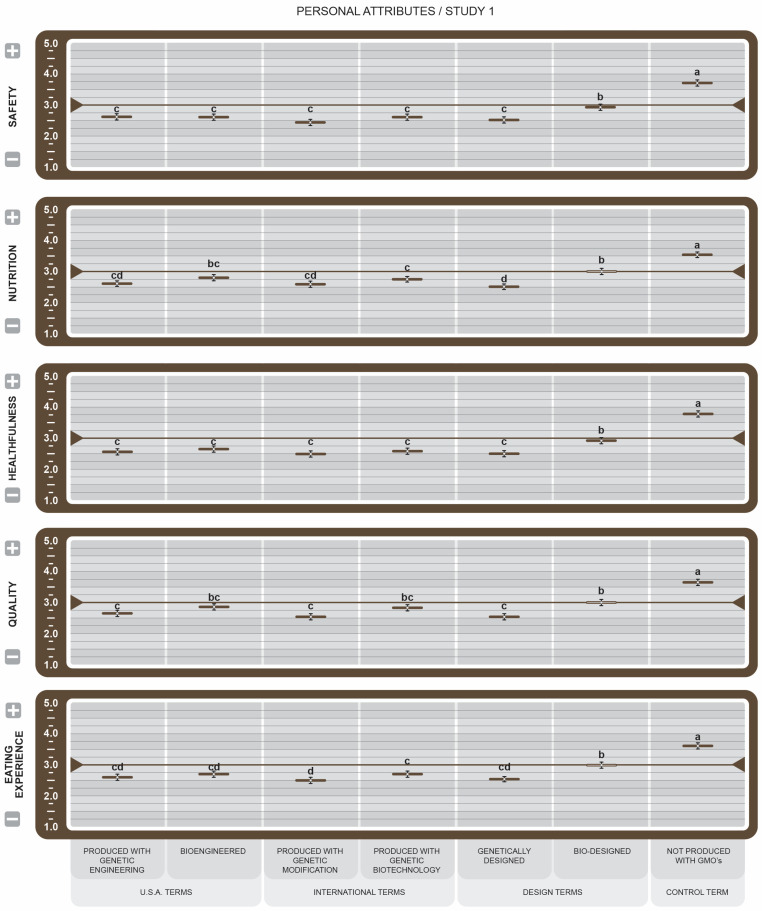
Study 1 effect of Genetically Engineered Disclosure statements on personal attributes. a–d: Like superscripts represent no significant differences between means (*p* < 0.05).

In personal attributes (see Figure 5), the mean scores for the control term were significantly higher than the other BDSs for Safety, Healthfulness, and Eating Experience and shared significance with *Bioengineered* in Nutrition and Quality. Furthermore, *Not Produced with GMOs* was the only label demonstrating substantially improved attitudes in this category. It is notable, however, that in this broad category of varied attributes, *Bio-designed* was the only BDS with consistently slightly positive scores ≥ the midpoint. As such, participants viewed it and *NGMO* labels as more safe, nutritious, healthful, and providing better quality and eating experiences. Conversely, scores for the remaining BDSs, i.e., *Generically Designed*, *Produced with Genetic Engineering*, and *Produced with Genetic Biotechnology,* were considered significantly negative.

#### 3.1.3. Study 1 Participant GMO Knowledge

The mean participant’s self-rated knowledge about GMOs was compared to the quantified scores of their written descriptions of GMOs. The mean self-rated knowledge of 2.89 (somewhat knowledgeable) was significantly higher than their mean quantified knowledge of 2.42 (slightly to somewhat knowledgeable), yielding an estimated difference between quantified and self-rated knowledge of 15% (*p* < 0.0001). Participants with a high school-level of education self-assessed their GMO knowledge on average 0.45 points higher than their quantified value (*p* < 0.05). Participants self-assessed versus quantified scores from the remaining eight education achievement levels were not significantly different. Participants who rated their eating habits as 1 (very unhealthy) gave themselves a GMO knowledge rating that was, on average, 0.79 lower than their quantified value. Those who rated themselves a 4 (somewhat healthy) rated themselves, on average, 0.24 higher than the quantified value (*p* = 0.04). Lastly, participants who gave themselves a 5 (very healthy) also rated their GMO knowledge to be, on average, 1.10 higher than the quantified value they received (*p* < 0.0001).

#### 3.1.4. Study 1 Participant GMO Importance

The majority (56.15%) of participants’ self-rated importance of GMOs was Somewhat, Slightly or Not at all important, as shown in Table 1.

### 3.2. Study 2 Results

#### 3.2.1. Study 2 Overall GMO Attitudes

**Figure 6:**
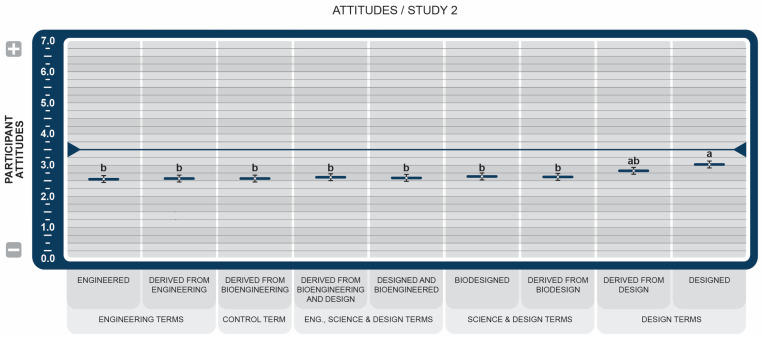
Study 2 effect of Genetically Engineered Disclosure statements on combined participant attitudes (positive/negative, like/dislike, favorable/unfavorable). a,b: Like superscripts represent no significant differences between means (*p* < 0.05).

Like Study 1, participants’ overall attitudes toward GM food products, as described by each BDS, were unfavorable, all scoring below the midpoint of 3.5. (see Figure 6).

The terms *Derived from Design* and *Designed* scored consistently higher than all other BDSs, including the control term, *Derived From Bioengineering*. Despite the low favorability scores, *Designed* scored significantly higher than the control term.

#### 3.2.2. Effect of BDSs on Food-Related Attributes

**Figure 7:**
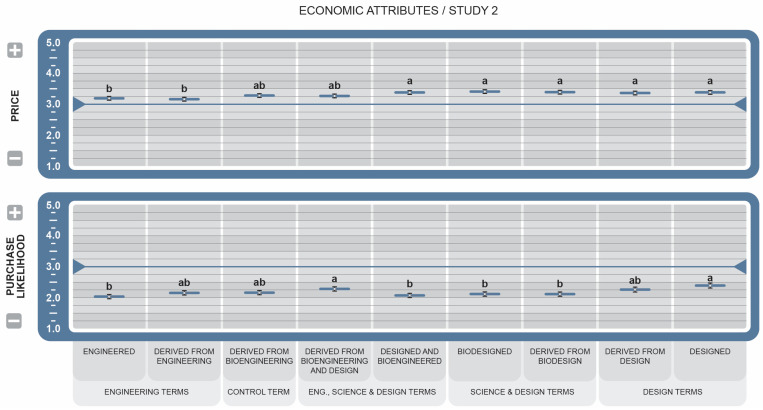
Effect of Genetically Engineered Disclosure statements on economic attributes. Means greater than the 3.0 midpoint were rated as increase/more likely to purchase, while those less than 3.0 were rated as decrease/less likely to purchase. a,b: Like superscripts represent no significant differences between means (*p* < 0.05).

Among the economic attributes (see Figure 7), the Price of all BDS-labeled foods was associated with higher prices, consistent with Study 1 findings. The BDS *Biodesigned* was rated as the most expensive (µ = 3.42 ± 0.07, *d* = 1.43). *Derived from Engineering* (µ = 3.17 ± 0.67, *d* = 2.23) and Engineered (µ = 3.20 ± 0.66, *d* = 1.80) terms were the most significant contributors toward attitudes of these higher costs. For Purchase Likelihood, all BDSs indicated decreased likeliness to purchase, consistent with Study 1. *Designed* (µ = 3.29, *d* = 2.75) and *Derived from Bioengineering and Design* (µ = 2.29 ± 0.07, *d* = 1.43) received the highest mean scores but were only directionally better than others.

**Figure 8:**
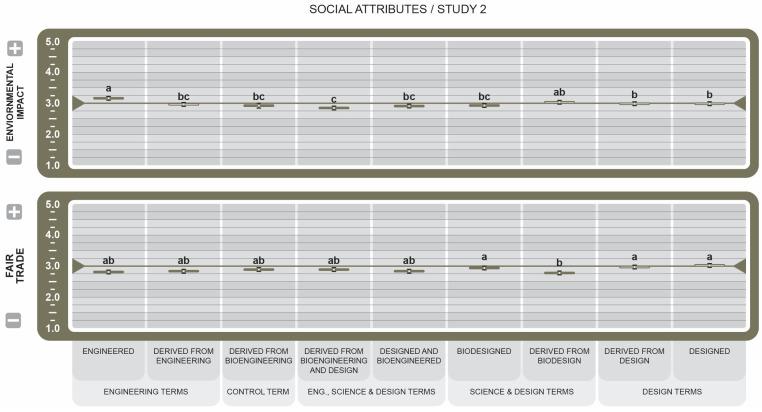
Effect of Genetically Engineered Disclosure statements on social attributes. a–c: Like superscripts represent no significant differences between means (*p* < 0.05).

Of the social attributes (see Figure 8), the BDSs *Designed* (µ = 3.03 ± 0.06, *d* = 2.35), *Derived from Biodesign* (µ = 2.78 ± 0.06, *d* = 1.78), and *Derived From Design* (µ = 3.0 ± 0.060, *d* = 1.42) were the primary drivers in influencing the positive perceptions of Fair Trade practices. While *Derived from Bioengineering and Design* (µ = 3.0 ± 0.06, *d* = 0.18) and *Derived from Bioengineering* (µ = 2.9 ± 0.06, *d* = 0.19) scored at parity with the two aforementioned BDSs, they had negligible influence on equitable production practices. For the attribute Environmental Impact, the BDS *Engineered* had the highest mean and size effect (µ = 3.11 ± 0.06, *d* = 2.0), indicating it has the greatest impact on positive perceptions of environmental responsibility and reduced environmental harm. *Derived from Bioengineering and Design* (µ = 3.0 ± 0.07) had the lowest score, indicating the least influence on environmental impact (*d* = 0.17).

**Figure 9:**
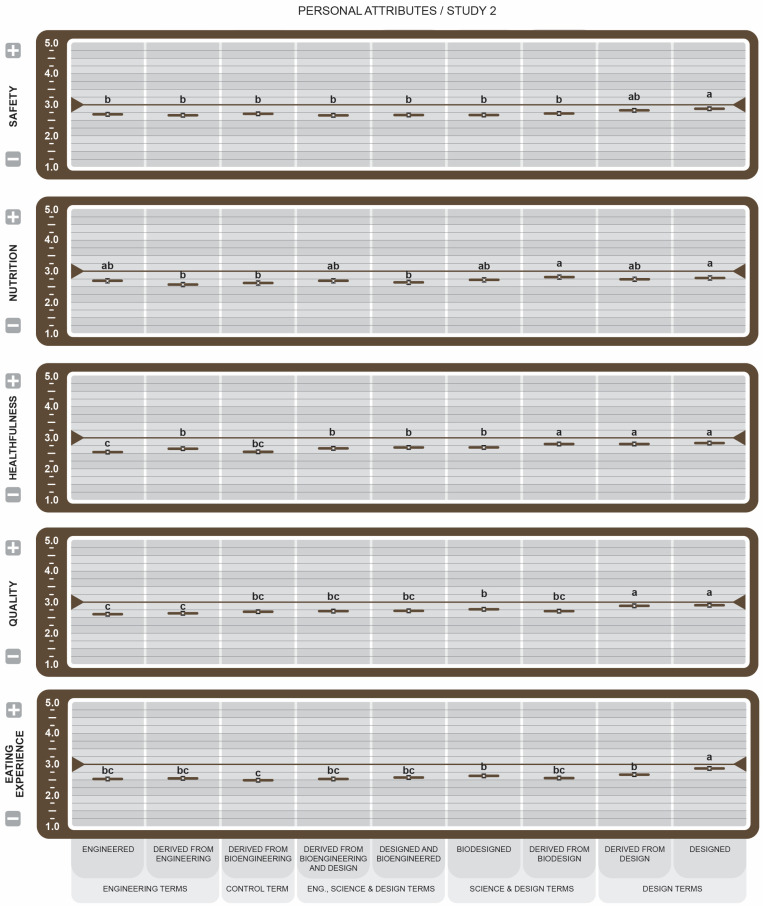
Study 2 effect of Genetically Engineered Disclosure statements on personal attributes. a–c: Like superscripts represent no significant differences between means (*p* < 0.05).

In the personal attributes category (see Figure 9), all BDSs scored below or slightly above the midpoint, supporting Study 1 results. This may suggest that as a group, GM-indicating messages on food may not be firmly aligned with these personal values or that consumers are indifferent. Leaning more positively toward the midpoint and, in many cases, scoring significantly higher than other BDSs, were *Designed* and *Derived from Biodesign*, suggesting that design-related labels may more closely support personal ideals.

**Figure 10:**
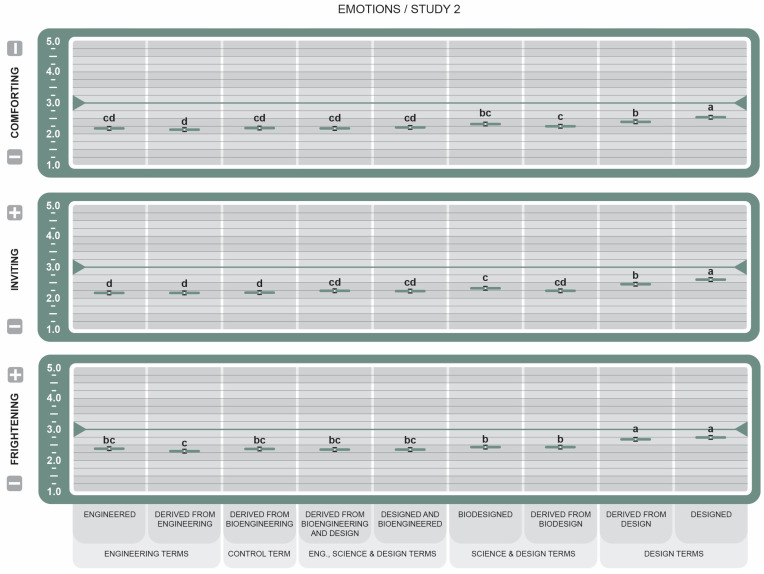
Effect of Genetically Engineered Disclosure statements on participant emotions. a–d: Like superscripts represent no significant differences between means (*p* < 0.05).

The results from the emotion attribute group (see Figure 10) conclude that *Designed* (µ = 2.55 ± 0.06, *d* = 4.45) had the greatest influence on comforting perceptions, while the terms *Derived from Design* (µ = 2.39 ± 0.06, *d* = 1.94), *Derived from Biodesign* (µ = 2.23 ± 0.06, *d* = 0.24), and *Biodesigned* (µ = 2.32 ± 0.06, *d* = 0.87), all BDSs that use design language, were also influential, though to a lesser but meaningful degree. Lower-scoring terms were those with the word root *Engineer*, which may indicate greater feelings of discomfort with engineering-focused terms. Similar trends were seen in the attribute Inviting. All BDSs elicited feelings of fright (lower scores mean more frightening), although significantly less so from *Designed* (µ = 2.75 ± 0.06, *d* = 4.62) and *Derived from Design* (µ = 2.43 ± 0.06, *d* = 3.68). *Derived from Engineering* (µ = 2.32 ± 0.06, *d* = 2.22) was deemed the most frightening.

**Figure 11:**
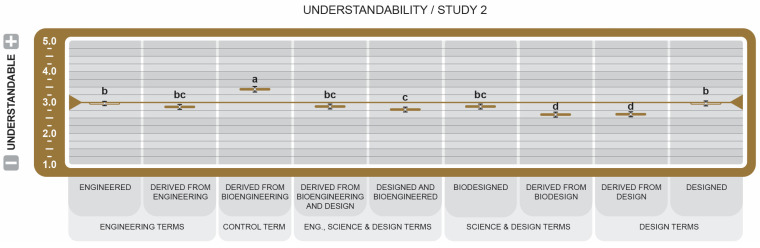
Effect of Genetically Engineered Disclosure statements on participant understandability. a–d: Like superscripts represent no significant differences between means (*p* < 0.05).

For the Understandability attribute (see Figure 11), *Derived from Bioengineering* (µ = 3.43 ± 0.09) was the most understandable of all other BDSs, with the only mean above the midpoint. Curiously, the BDSs *Derived from Design* (µ = 2.62 ± 0.07) and *Derived from Biodesign* (µ = 2.61 ± 0.07) were significantly less understandable than the other BDSs, despite their rating being significantly more favorable for the majority of attributes.

**Figure 12:**
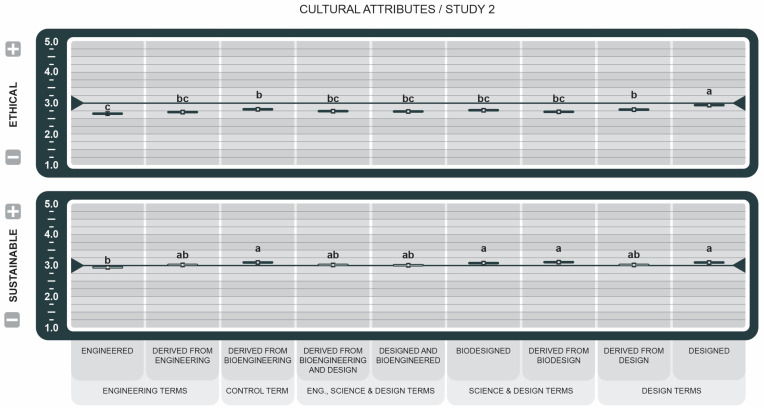
Effect of Genetically Engineered Disclosure statements on cultural attributes. a–c: Like superscripts represent no significant differences between means (*p* < 0.05).

Among the cultural attributes (see Figure 12), *Designed* (*d* = 2.79), *Derived from Biodesign* (*d* = 1.12), and *Derived from Engineering* (0.80) had positive associations and alignment with ethics. *Engineered* scored significantly lower and, based on its very large effect (*d* = 1.72), consumers are likely to associate it with low integrity. *Engineered* had the lowest mean and the largest negative associations (*d* = 1.61) related to sustaining responsible production. Conversely, *Derived from Biodesign* (*d* = 1.12), *Derived from Bioengineering* (*d* = 0.93), and *Designed* (*d* = 0.95) were among the significantly higher means, and showed the greatest impact on conveying high sustainability.

## 4. Discussion

Across both studies, design-related labels such as *Designed* and *Bio-designed* consistently outperformed traditional engineering/science-based terms such as *Genetically Modified* and *Engineered*, and fostered positive perceptions. While design-focused terms show potential for a positive impact, even the best-performing design-language labels did not fully overcome negative contamination from the entrenched skepticism associated with GM foods, underscoring the need for complementary strategies beyond linguistic changes.

### 4.1. Study 1 Discussion

#### 4.1.1. Study 1 Overall Attitudes

Participants’ overall attitudes toward all BDSs were significantly lower than the control term, *NGMO* (see Figure 2). This finding is consistent with the results reported by Lefebvre et al. [39], who found that consumer opinions of products that included a GM disclosure declined significantly compared to the same product with no disclosure, or an organic one. The new term, *Bio-designed*, had a significantly more positive effect on consumer attitudes than all other BDSs, except *Bioengineered,* and was the only BDS that did not score below the midpoint rating. This result was the impetus for further exploration of the more human-centered semantics of design, the scientific associations with bio, and the word engineering explored in Study 2.

#### 4.1.2. Assessing BDS Component Words

The component words for each BDS, *Design*, *Bio*, *Genetic*, *Engineer*, *Technology*, *Produced*, and *Modification*, were not statistically assessed for effect on attitudes. However, averaging cumulative ratings indicates consumers might respond more favorably to specific root words. While not evaluated for significance, *Design* and *Bio* had an average score of 3.16 and 3.14. *Engineering* and *Technology* had an average score of 2.85 and 2.82. *Modification*, *Genetic*, and *Produced* scored at 2.72, 2.71 and 2.61. The positive leaning results of *Design* and *Bio* were a second impetus to explore more words in Study 2. In a study by Porcar et al. [40], the term “biotechnology” scored the highest, while “synthetic biology” scored the lowest, indicating the importance of root words in a disclosure statement.

#### 4.1.3. Effect of BDSs on Food-Related Attributes

The pairing of the BDS plus the control term with the nine food-related attributes generally mirrored consumers’ overall attitude toward GM foods. In economic attributes of Price and Purchase Likelihood, (see Figure 3), survey participants felt that GM food costs would increase when labeled with any of the BDSs and that the cost of food bearing the control term would increase significantly more than all other BDSs, followed by *Bio-Designed*. Several studies in the US and abroad have investigated the effect of labeling foods as not containing GMOs [41,42,43], with mixed results as to its importance in purchase decision-making. In this study, the positive Purchase Likelihood ratings for *Bio-Designed* suggest that GM/BE foods bearing this label may be perceived as having greater value and might nudge consumer perceptions toward an expectation of higher cost. The two BDSs that performed most positively after the *NGMO* label included *Bio* as a prefix, which may also carry some merit for future exploration in commercial GM/BE food communication, suggesting that some words are associated with being less expensive.

The fact that participants declared a greater willingness to purchase foods bearing *NGMO* and *Bio-Designed* labels (see Figure 3), even when they considered that such food products would be higher-priced, indicates the economic power given to *NGMO*-labeled products and other descriptors that contribute positive contamination. This possibility is supported by the works of Kim et al. [44] and Hu et al. [45]. These results undermine previous research on “purchase likelihood” from Heslop [46], whose study found minimal GM labeling effects overall. One explanation for the present results may be that consumer perception of GM products has been shaped in the last 10 to 15 years by increased media attention and the organic food industry’s marketing efforts [47]. These results also suggest that there is power to influence action when the right words are used. Consequently, a strategically worded BDS could be used as a marketing advantage. Given that Price and Purchase Likelihood (see Figure 3) represented the greatest extremes in effect in the study’s collection of questions, consumers appear to recognize the potential economic implications of GM/BE foods when weighed against social or personal attribute values.

In social attributes (see Figure 4), the effect ratings for Fair Trade and Environmental Impact revealed consumer perceptions that foods labeled *NGMO* would significantly increase responsible trade opportunities and decrease impact on the environment. While the effect of *Bio-Designed* on these attributes is lower than the control term, its effect is positive. In contrast, products bearing all BDS labels would decrease Fair Trade perceptions and increase Environmental Impact. These results are consistent with the reported findings of Han and Harrison [48], who found that ethical and environmental concerns were the primary motivators in consumers’ choice not to purchase food from GM sources.

In personal attributes (see Figure 5), ratings for Safety, Nutrition, Healthfulness, Quality, and Eating Experience revealed generally matching results. Survey participants felt that foods bearing any of the proposed BDS besides *Bio-Designed* would be less safe, nutritious, and healthy, of lesser quality, and provide a less favorable eating experience than foods that display this more positively viewed BDS. These results are not surprising due to decades of persistent consumer caution and negative perceptions of GM designations. The influence of negative contamination in this attribute category supports the findings that humans give greater credence to negative over positive information and that negative associations remain on top of the mind longer than their counterparts [49]. Furthermore, consumers have been shown to overact when news is negative, and such sentiment is exacerbated by collective public exposure [50]. Our finding that *NGMO* received significantly higher ratings than the BDS is consistent with previous studies reporting that consumers perceive products without GM ingredients to be healthier than products that contain GM ingredients [51]. The Nutrition and Quality (see Figure 5) attributes for BDS-labeled foods performed slightly better with consumers than Healthfulness, Safety, and Eating Experience. Entities that position GM food products from a nutrition or quality perspective may generate marginally better appeal among their consumers than positioning them as healthy, safe, or experiential. In contrast to this finding, Han and Harrison [48] reported that among consumers willing to purchase foods from GM sources, their belief in the inherent safety of the foods was the primary motivator.

Across the economic, social, and personal attributes (see Figure 3, Figure 4 and Figure 5), the consistent favorability for the control term supports the notion that the non-GMO movement grew out of the initial controversy from the first commercially available GMO foods in the 1990s. The two dominant label standards today are the non-GMO project’s “Non-GMO Project Verified” butterfly label and the USDA National Organic Program’s (NOP) “USDA Organic” seal [52,53]. Ultimately, *NGMO* was favored in all attribute ratings, with *Bio-Designed* as the next most positive in every category. The long-term fate of GM/BE foods due to the non-GMO movement appears established. Evidence shows that non-GMO messaging, present in the lexicon for three decades, enjoys a positive interaction with consumers.

#### 4.1.4. GMO Knowledge and Importance

Terminal education through high school was the only variable that showed significance between consumers’ self-rated and actual GMO knowledge. This finding adds another perspective to the questionnaire-based studies of Harrison et al. [54] and Puduri et al. [55], who found that consumers with a high school diploma were less willing to purchase GM foods than consumers with less and higher education. Zheng and Wang [42] found that 86% of those surveyed self-reported having little or no GMO knowledge, while their accuracy in answering specific questions about them was as high as 85%. From this, they hypothesized that consumers’ perceptions of GMOs are more influential than their accurate understanding of them. Wunderlich and Gatto [56] concluded that a distinction between self-reported familiarity with GMOs and a scientific understanding of them was needed. The former could be described as “GMO knowledge”, as phrased in the present study; however, the latter involves a more in-depth knowledge of its principles. A distinction should be made between these two states of understanding, as consumers whose self-assessed GMO knowledge was higher than their accurate understanding were more resistant to GMO foods [57]. One explanation may be that those with greater knowledge of the subject tend to have fewer negative attitudes toward GMO foods [56,58]. It is notable, however, that having participants express their understanding of GMOs in writing may not be comprehensive due to individual differences in communication styles and strength [59].

### 4.2. Study 2

Study 2 revealed an even stronger performance of design-related labels across economic, personal, social, emotional, and cultural attributes in support of Hypothesis 2 that design terms would be viewed positively, while mitigating general skepticism toward GM foods. While the results for design terms lean positively toward more neutral or even positive attitudes, we learned that language alone is not enough to overturn prevailing negative beliefs.

#### 4.2.1. Study 2 Overall Attitudes

All BDSs, including the control statement, in Study 2 did not score at or above the midpoint of 3.5 (see Figure 6), which further supports our findings from Study 1 that the overall consumer attitudes favor foods that explicitly state that they do not contain GM/BE components. However, the BDS *Design* consistently scored the highest and scored significantly higher than the engineering- and science-based terms across multiple scales. Participants were more likely to respond positively to *Design* within the context of a BDS, demonstrating the ability of specific words on food labels to nudge consumers directionally.

#### 4.2.2. Study 2 Effect of BDSs on Attributes

For economic attributes (see Figure 7), participants felt that foods labeled with any BDS would be sold at a fair price unlike participants in Study 1. This could suggest a change in consumer attitudes toward GM/BE foods concerning cost during the time between the two studies. Regardless of their attitudes, consumers may have come to believe that GM/BE foods can help reduce food costs compared to their non-GM counterparts [60]. Overall, consumer perception that labels disclosing the inclusion or absence of GMOs will increase costs is likely accurate. According to one study, the economic implications of the new federal law are expected to be worse than having no law, but better than the inconsistency of multiple different state laws [61]. Foods not required to bear a GM/BE label or that choose to display a “free-from” label, such as *NGMO*, will require verification, monitoring, record keeping, and perhaps the segregation of products, all of which increase costs, ultimately making policies related to labeling and identification even more critical.

Among social attributes (see Figure 8), it is notable that engineering terms scored slightly lower on average regarding Fair Trade and significantly higher regarding Environmental Impact. Nearly all BDSs scored at the midpoint, which could imply either misunderstanding or disassociation between the BDSs presented and the two different attributes. It could be valuable to investigate this category further, focusing more on the relationships between the social knowledge of Fair Trade, Environmental Impact, Sustainability, and the related BDSs. Additionally, among all the groups of labeling terms, the design term group scored consistently more favorable. This is worth noting in conjunction with the ongoing effort to help the public understand the importance of GMOs in the greater food landscape. Associations and principles of design could be used to help ground and unify an understanding of GMOs.

For personal attributes (see Figure 9), survey participants reported that all foods bearing any of the proposed BDSs would provide less Safety, Nutrition, Healthfulness, Quality, and a less favorable Eating Experience when compared with the *NGMO* score in Study 1. Assessing the control and engineering terms against the design terms, scores indicate that for Healthfulness and Quality attributes, *Designed* GM/BE food products are significantly preferred over *Engineered* food products. They also broadly considered *Designed* foods more Safe, Nutritious and a better Eating Experience than *Engineered* foods. Thus, manufacturers who position GM food products from a *Designed* rather than an *Engineered* perspective may increase their appeal among consumers.

For emotion attributes (see Figure 10), all BDSs were scored less than favorable. Several studies report that GM-related terms, such as “genetically modified”, elicit confusion and hesitation in consumers [58,62,63,64,65]. Additionally, one study cited in the FDA discussion accompanying the NBFDS final rule states that interpretations of “Bioengineered” labels were inconsistent, unclear, and often related to warning or caution [66]. While developing new technology, decisions are often made by scientists and engineers who are experts in emerging technology but likely have a limited understanding of human behavior, which is incredibly complex. Technology experts can mistakenly believe that knowledge and logic alone are sufficient for a good design outcome [37] and disregard the emotional influence of consumer behavior. Critics of rational choice models like the Theory of Reasoned Action (TRA) and the Theory of Planned Behavior (TPB) have argued that individuals do not typically behave in a rational way, and may be guided by more emotive, moral, and altruistic principles [67]. The design terms were viewed as significantly more Inviting, Comforting, and less Frightening (see Figure 10) than the science and engineering terms. The small but significant differences in attitudes suggest again that word choice of GM/BE labeling matters in terms of emotions.

For Understandability (see Figure 11), *Derived from Bioengineering*, scored significantly higher than all other BDSs, but not high enough to be considered commonly understandable. One explanation could be that public exposure to a consistent, standardized statement may have increased its familiarity with consumers but not their understanding. There is ultimately a significant difference between awareness and education level. These findings acknowledge, however, that the differences in understanding are due to other contributors such as context, affect [68], and cultural differences. Quesque et al.’s international cross-culture study supports this influence as significant in understanding the thoughts and emotions of others [69]. Additionally, while the control term was rated as more understandable, this did not translate to the statement being more favorable in other attributes. This may suggest that the use of comprehensible words is critical, and labeling compliance in accordance with legislative requirements is no assurance that they will be broadly understood [61].

For the cultural attributes (see Figure 12), all tested BDSs were rated below the midpoint in the Ethical attribute, but just above midpoint for Sustainability, except for the BDS *Engineered*, which was again scored as being the least sustainable BDS. Despite the numerous advantages of GM technology [70], public perception of potential human allergenicity and toxicological risks, and environmental harm still outweigh the less visible benefits [71]. Since sustainability is an important value, this positive viewpoint, among the continued negative feelings, could be a feature that manufacturers and sellers capitalize on to nudge consumer acceptance. This presents an exciting finding within our investigation: consumers, despite viewing specific BDS-labeled GMO food products as both less ethical and having negative environmental impacts, also believe GMO food products to be sustainable.

### 4.3. Future Studies

**Figure 13:**
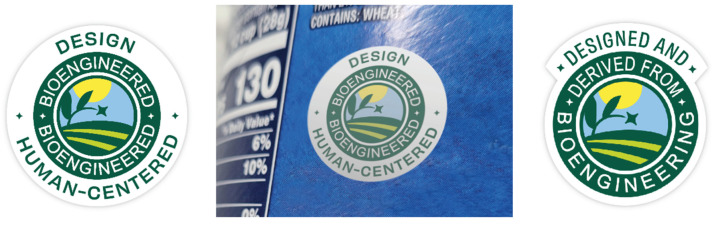
The green colored labels with white Bioengineered and Derived from Bioengineering type are the USDA authorized regulatory stickers. The white backgrounds with green type stating “Design, Human-Centered” and “Designed And” are proposed additions combining design signifiers with the required labels. The center image is a proposed label placed on an existing package using photoshop.

Further research should consider labeling within specific contexts and cultures, which influences the perception and understanding of GM/BE technology. Expecting current disclosure language to evolve might be wishful; however, testing known positively contaminated signifiers like *Design* in combination with regulated label terms might improve GM/BE food perceptions, for example, *Design*, *Human-Centered*, *Bioengineered*, or *Designed and Derived from Bioengineering* (see Figure 13).

### 4.4. Limitations of the Current Study

This study’s limitations may reduce the generalizability of its findings. First, while convenience sampling allowed for the collection of a large dataset, it may not fully capture the diversity of consumer perceptions across demographic groups. Future research should employ stratified, random, or cross-cultural sampling methods to ensure a more representative participant pool, incorporating factors such as age, education level, cultural background, and socioeconomic status. Likewise, while this study hypothesized that “design” would be perceived more favorably than “engineered” or “genetic”, these terms may not carry universal connotations across cultural and linguistic contexts, particularly about food.

The study did not assess subjects’ prior knowledge of or preexisting attitudes to-ward current or newly introduced BDS terminology, including control terms. Measuring baseline knowledge and attitudes toward GM food labeling language could control for these confounding factors and provide a clearer understanding of how terminology influences perceptions. External factors, such as prior experiences with GM foods and media exposure, may influence consumer responses beyond linguistic framing alone. Consumer attitudes toward GMOs are often deeply ingrained, meaning that survey-based responses may be shaped by preexisting biases rather than objective evaluations of labeling terminology. Future studies should consider accounting for such variables. Furthermore, relying solely on free response writing to evaluate self-reported GMO knowledge may not fully capture comprehension due to differences in communication strengths. Complementary methodologies, such as structured or multiple-choice questions, could improve the consistency and accuracy of knowledge. Beyond linguistic considerations, the study does not fully explore the psychological mechanisms through which specific terms influence perceptions of characteristics such as safety, healthfulness, sustainability, and emotions. Integrating established cognitive and affective consumer decision-making models could provide a deeper understanding of why particular terms evoke more positive or negative responses.

## 5. Conclusions

In this study, we hypothesized that the science- and engineering-discipline-based words currently used in GM food disclosure statements are negatively contaminated by persistent unfavorable messages that influence consumer perceptions of GM foods. Additionally, we hypothesized that introducing the signifier *Design* with similar semantic meanings but unassociated with science and engineering values could mitigate the effects of negatively contaminated consumer perceptions of GM foods.

This study shows that additional descriptor phrases or statements did not markedly alter consumers’ attitudes toward GM/BE foods. However, the results reveal significant and practical differences in consumer perceptions of GM/BE foods depending on the labeling terminology used. The findings in Study 1 revealed that engineering/science-based labels are deeply associated with negative consumer attitudes and that design-focused labels evoked positive associations. Besides the control term, all BDSs, except *Bio-designed*, had a less than favorable impact on the participant’s perception of all attributes and had the greatest influence on positive perceptions. Consumers appear to associate most engineering/science-based terms with concerns in all attribute categories. Study 2 revealed an even more potent performance of design-related labels across economic, personal, social, emotional, and cultural attributes compared to engineering- and science-based terms. Specifically, the distinct score differences in the Comforting, Inviting, and Ethical attributes suggest a lack of trust in the current disciplinary lexicon and an opportunity for stakeholders to nudge perceptions by understanding consumers’ emotional and ethical aspirations. For example, positively contaminated content can be explored in association with existing labels by uniting a design signifier with a current regulatory label.

As longstanding consumer perceptions of GM foods are skewed toward aversion, fearfulness, and skepticism, addressing disclosure terms alone is insufficient to overturn prevailing negative perceptions of GM foods. However, design terms significantly improved consumer perceptions over the currently legislated BDSs, making their implications in the context of labeling and statement construction meaningful. Future research should include survey methodologies that reach more diverse populations and consider the breadth of confounding variables that may skew results. Finally, expanding research to encompass various food commodities such as fresh produce, grains, and animal-derived products would offer more understanding of how disclosure statements influence consumer choices.

## Figures and Tables

**Table 1:** Participants’ responses to the question “How important is the topic of GMOs to you?”.

Importance	Total Participants	Percentage of Participants
Extremely important	150	11.32%
Very important	212	16.00%
Moderately important	219	16.53%
Somewhat important	291	21.96%
Slightly important	267	20.15%
Not at all important	186	14.04%
Total	1325	100%

## Data Availability

The original contributions presented in the study are included in the article, further inquiries can be directed to the corresponding author.
